# Selective Kanji agraphia in crossed aphasia after right-hemisphere infarction: a case report

**DOI:** 10.1590/1980-5764-DN-2025-0464

**Published:** 2026-06-15

**Authors:** Takahiro Kowari

**Affiliations:** 1 Kawasaki University of Medical Welfare, Faculty of Rehabilitation, Department of Speech-Language-Hearing Therapy, Kurashiki, Okayama, Japan.

**Keywords:** Aphasia, Agraphia, Dominance, Cerebral, Infarction, Posterior Cerebral Artery, Afasia, Agrafia, Dominância Cerebral, Infarto da Artéria Cerebral Posterior

## Abstract

Crossed aphasia is a rare form of aphasia in right-handed individuals with right-hemisphere lesions. We report a man aged 67 years who developed crossed aphasia after a right posterior cerebral artery infarction involving the occipital lobe, thalamus, and medial parietal lobe. Neurological and neuropsychological examinations revealed mild anomic aphasia with preserved repetition and comprehension, left homonymous hemianopia, unilateral spatial neglect, prosopagnosia, and no apraxia. Writing showed a marked script-specific dissociation: kana writing was intact, whereas kanji writing was severely impaired and dominated by recall-failure errors (>90%), with few orthographic or component errors. One-year follow-up using the same language and writing tasks demonstrated partial improvement in accuracy but a stable error profile dominated by recall failures. This rare case indicates that selective kanji agraphia can occur in crossed aphasia due to right-hemisphere lesions and suggests impaired access to the visual orthographic lexicon with atypical hemispheric lateralization of writing functions in Japanese.

## RESUMO

A afasia cruzada é uma forma rara de afasia em indivíduos destros com lesões no hemisfério direito. Relatamos um homem de 67 anos que desenvolveu afasia cruzada após infarto da artéria cerebral posterior direita envolvendo o lobo occipital, o tálamo e a região parietal medial. Os exames neurológico e neuropsicológico mostraram afasia anômica leve com repetição e compreensão preservadas, hemianopsia homônima esquerda, negligência espacial unilateral e prosopagnosia, sem apraxia. A escrita evidenciou dissociação clara entre sistemas: kana preservado, enquanto kanji estava gravemente prejudicado e dominado por erros de falha de evocação (>90%). A reavaliação após um ano com as mesmas tarefas demonstrou melhora parcial, mas manutenção do perfil de erros dominado por falhas de evocação. Este caso sugere agrafia seletiva para kanji na afasia cruzada por lesão do hemisfério direito, associada a acesso prejudicado ao léxico ortográfico visual.

## INTRODUCTION

Crossed aphasia is a rare aphasia that occurs in right-handed individuals following right-hemisphere brain damage, accounting for 1–3% of all aphasia cases^1^. Reported writing disturbances include jargon agraphia^2,3^, kana/kanji recall failure after right frontal periventricular lesions^4^, and orthographic errors in kanji following right putaminal lesions^5^. However, kanji agraphia in crossed aphasia remains poorly characterized because the reported cases are few and heterogeneous in lesion location and error profile^6^.

The Japanese writing system comprises two scripts with distinct properties: kana, a phonetic syllabary in which each character corresponds to a syllable and carries no inherent meaning, and kanji, a logographic script whose characters convey meaning as well as phonology. Kana writing is thought to rely mainly on phonological (non-lexical) pathways, whereas kanji writing depends more on lexical-orthographic processing involving the left posterior inferior temporal cortex and bilateral cortical regions^7,8^. Script-specific dissociations in crossed aphasia may therefore provide insight into the neural basis and hemispheric lateralization of writing.

Most reported kanji writing disorders after right-hemisphere damage have been attributed to visuospatial or attention-related agraphia^9,10,11^, whereas cases dominated by kanji recall failure with preserved kana writing are rare^10,11^.

The present case involves crossed aphasia caused by lesions of the right occipital lobe, thalamus, and medial parietal surface, with word-finding difficulty, kanji agraphia, left spatial neglect, and prosopagnosia. We focus on whether kanji recall failure reflects impaired access to the visual orthographic lexicon, how it relates to right occipitothalamic/parietal networks, and why alexia did not occur despite aphasia.

## CASE REPORT

### Clinical history

A strongly right-handed Japanese man aged 67 years (Edinburgh Handedness Inventory score 100), with no family history of left-handedness and 12 years of formal education, was a monolingual native Japanese speaker. On day 1, he experienced a seizure, after which he had difficulty operating his cell phone and was disoriented. He presented to the emergency department and was admitted on the same day. The initial neuropsychological assessment was conducted during the acute inpatient phase (days 10–16), and the follow-up took place about one year later (day 373) in an outpatient stroke clinic.

### Neuroimaging findings

**Figure 1:**
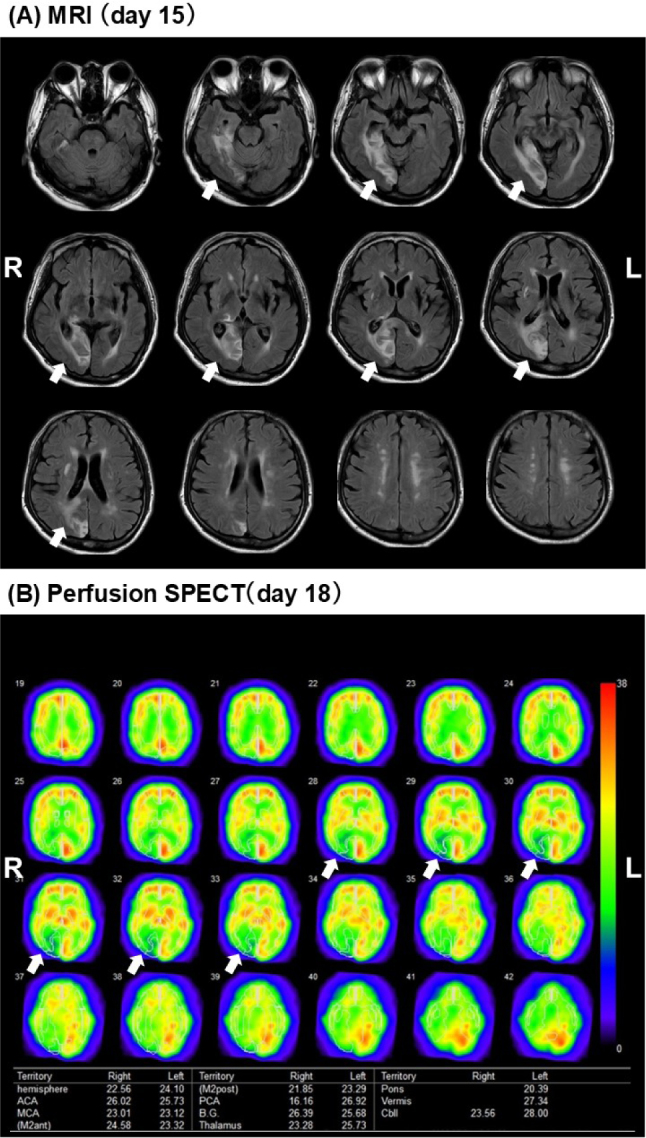
Structural magnetic resonance imaging — MRI (day 15) and perfusion single photon emission computed tomography — SPECT (day 18). MRI shows high-signal lesions in the right thalamus, medial temporal lobe, medial parietal lobe, and occipital lobe. SPECT demonstrates hypoperfusion within the right posterior cerebral artery territory and adjacent regions.

Magnetic resonance imaging (MRI) on day 15 demonstrated high-signal lesions in the right occipital lobe, thalamus, medial temporal lobe, and medial parietal lobe, corresponding to the right posterior cerebral artery territory. Although the day-15 fluid-attenuated inversion recovery (FLAIR) and diffusion-weighted imaging (DWI) images were reviewed, splenial involvement was not definite. Perfusion single photon emission computed tomography — SPECT (day 18) revealed hypoperfusion predominantly in the right PCA territory and adjacent regions (Figure 1).

### Neurological examination

There was no motor paralysis or denervation; muscle tone and tendon reflexes were normal. He had left homonymous hemianopia. No limb or oral apraxia was observed, and there was no topographical disorientation in navigation within the hospital or familiar environments.

### Neuropsychological findings

Spontaneous conversation showed word-finding difficulty and reduced comprehension with increasing information load. During testing, delayed responses to left-sided stimuli and omissions on figure copying suggested left spatial neglect with constructional disorder. After ≈20 minutes, sustained attention declined, with calculation errors and omissions online cancellation. Prosopagnosia was evident (no sense of familiarity for the therapist’s face). On standardized language testing, the profile was fluent, mild anomic aphasia with preserved repetition and comprehension. A salient feature was disproportionately severe kanji agraphia relative to overall aphasia.

### Neuropsychological assessment

While inpatient (days 10–16), language functions were assessed with the Standard Language Test of Aphasia (SLTA) and the Sophia Analysis of Language in Aphasia (SALA), including D38 Word Writing — kanji (imagery × frequency) and D39 Word Writing (notation type × number of morae). Cognitive assessments included Mini-Mental State Examination — Japanese (MMSE-J), Raven’s Coloured Progressive Matrices (RCPM), Visual Perception Test for Agnosia (VPTA), Behavioral Inattention Test (BIT), and Rey-Osterrieth Complex Figure (ROCFT).

Incorrect kanji responses were classified into three types, as previously described^12^: orthographic errors (substitution with visually similar kanji), component errors (addition, omission or misplacement of radicals/strokes), and recall failures (inability to retrieve the target character). Each error type was coded for every kanji tested^12^. In addition, to examine whether the writing impairment could be influenced by spatial agraphia associated with right-hemisphere damage, spatial features of written products (e.g., systematic left-lateralized omissions of radicals/strokes, spatial deviation or misalignment within the character frame, and baseline drift in sentence writing) were independently coded based on previous descriptions^13,14^.

At day 373, a reappraisal was performed using the same SLTA and SALA tasks to examine change over time.

### Ethics

This report was approved by the Ethics Committee of Kawasaki Medical School Hospital (Approval No. 6784-00). Written informed consent for publication (including de-identified clinical, neuropsychological, imaging, and handwriting data) was obtained.

## RESULTS

**Figure 2:**
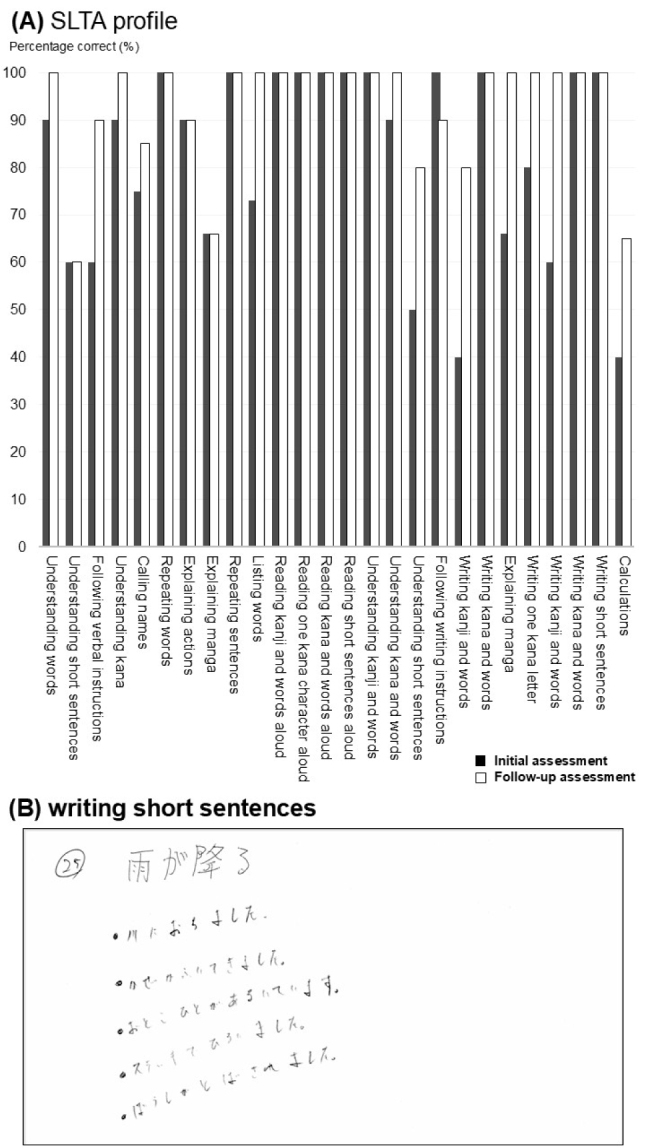
Standard Language Test of Aphasia — SLTA profile (initial assessment and 1-year follow-up) and representative examples of SLTA sentence dictation. SLTA subtest performance and representative sentence-dictation samples obtained at the initial assessment (days 10–16) and at the follow-up evaluation (day 373). In sentence dictation, the writing baseline was maintained horizontally, and no rightward drift on the page was observed.

On the initial SLTA (days 10–16), spontaneous speech and naming were impaired, whereas repetition and reading aloud were preserved. Sentence-level comprehension was preserved, and kanji writing/dictation showed persistent recall failure that did not respond to cueing. The SLTA profile and sentence-writing sample at the initial and follow-up evaluations are shown in Figure 2, and the results of the neuropsychological tests are summarized in Table 1.

**Table 1:** The results of the neuropsychological tests (initial days 10–16; follow-up day 373).

Domain	Subtest		Initial assessment	Follow-up assessment
SALA writing tasks	D38 word writing Kanji (imagery x frequency)	Kanji	15/48	29/48
	D39 word writing Kanji (notation type x number of mora)	Hiragana	30/30	30/30
		Katakana	30/30	30/30
		Kanji	14/30	18/30
MMSE-J			26/30	NA
RCPM			15/36	NA
ROCFT			10/36	NA
BIT	Conventional subtests		80/146	NA
	Line crossing		24/36	NA
	Letter cancellation		22/40	NA
	Star cancellation		30/54	NA
	Figure and shape copying		1/4	NA
	Line bisection		3/9	NA
	Representational drawing		0/9	NA
	Behavioral subtests		52/81	NA
	Picture scanning		1/9	NA
	Telephone dialing		9/9	NA
	Menu reading		3/9	NA
	Article reading		1/9	NA
	Telling and setting the time		5/9	NA
	Coin sorting		9/9	NA
	Address and sentence copying		9/9	NA
	Map navigation		9/9	NA
	Card sorting		6/9	NA

Abbreviation: NA, not assessed at follow-up. Note: Scores indicate number correct/total.

**Figure 3:**
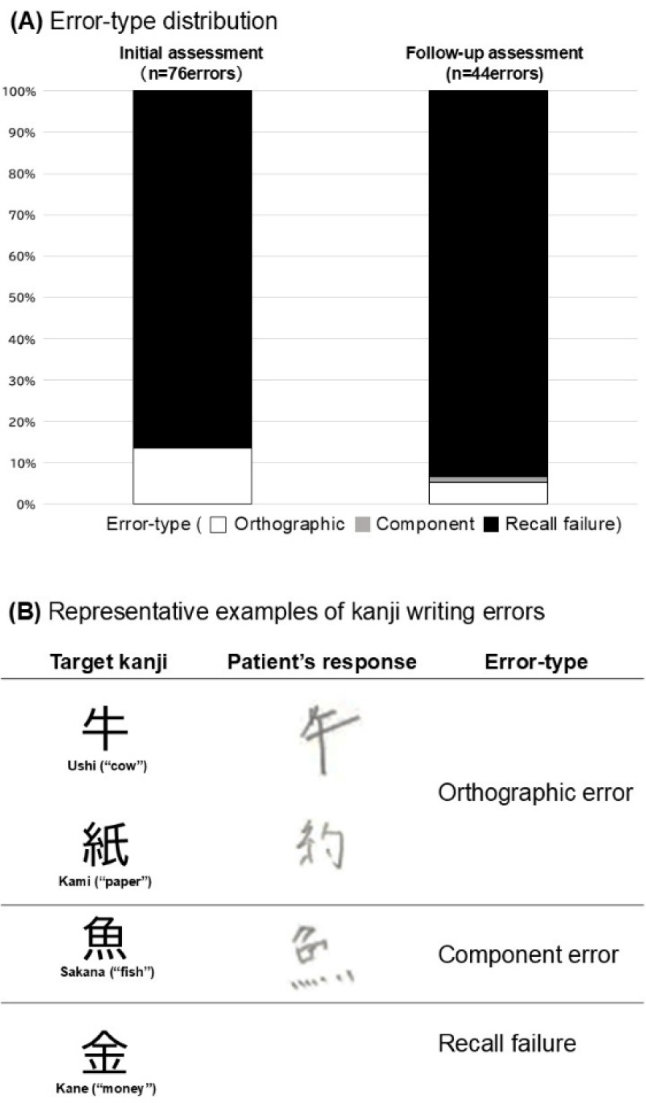
Distribution of kanji error types and representative examples. (A) Error-type distribution (recall failure, orthographic, component) at initial and follow-up assessments. (B) De-identified examples for each error type; patient consent obtained.

On SALA, D38 (kanji) yielded 15/48 (31.3%), with perfect performance on D39-hiragana and D39-katakana; D39-kanji was 14/30 (46.7%). The kanji error distribution was: recall failures 71/76 (93.4%), orthographic errors 4/76 (5.3%), and component errors 1/76 (1.3%). No abnormalities in writing sequence or penmanship were observed. In addition, no spatial features of written products were observed at the initial assessment; specifically, such features were observed in 0/76 kanji error responses, and the sentence-writing baseline was maintained horizontally (Figure 2). Error distribution and representative examples are shown in Figure 3.

Other neuropsychological results were as follows: MMSE-J 26/30; RCPM 15/36; ROCFT 10/36 with left-sided omissions and visuospatial distortions. On the BIT, 11 of 15 subtests were below the cut-off, indicating visuospatial impairment; however, address and sentence copying was preserved (9/9) (Table 1). On VPTA, performance was reduced on face-related subtests requiring identification by pointing or matching; detailed VPTA results are provided in Supplementary Material Table S1 — available at https://www.demneuropsy.org/wp-content/uploads/2026/03/DN-2025.0464-Supplementary-Material.pdf.

At the one-year reappraisal (day 373), language function improved. On the SLTA, writing and copying kanji words were 5/5 each. SALA D38 improved to 29/48 (60.4%), and D39-kanji to 18/30 (60.0%). However, the error profile remained similar: recall failures 38/44 (86.4%) and orthographic errors 6/44 (13.6%). No speech and language therapy was provided between the initial and follow-up evaluations. Because the follow-up assessment was limited to a single day, non-language neuropsychological tests, including the BIT, were not administered at follow-up.

## DISCUSSION

This case involved a right-handed patient who developed crossed aphasia due to a right-hemisphere lesion. Although aphasia was mild, it was characterized by prominent recall failures in writing kanji. The clinical picture — preserved kana writing and absence of alexia — demonstrated a script dissociation. Key points:

- kanji-specific agraphia with recall-failure predominance occurred after right PCA infarction;
- the error distribution remained stable over one year despite partial gains in accuracy; and
- preserved kana writing and reading argue against a global graphemic output or phonological deficit.

### Characteristics of writing in this case

In addition to word-finding difficulty, the patient showed a selective kanji writing disorder. Kana writing remained intact, as indicated by unchanged SALA D39 hiragana/katakana scores, whereas D38 and D39 kanji tasks were clearly impaired. In the acute phase, over 90% of kanji errors were recall failures, with only a few orthographic and component errors; at one-year follow-up, overall accuracy improved but the relative error distribution was similar (Figure 3).

Compared with previous crossed-aphasia cases reporting kanji agraphia^10,11^, the persistence of this error profile and the strong predominance of recall-failure errors are distinctive. Marked stroke distortions, spatial misalignment, or crowding — typical of visuospatial agraphia — were rare, making it unlikely that the writing disorder was primarily driven by visuospatial or attentional deficits, despite the presence of neglect and constructional disturbance.

### Mechanisms underlying kanji recall failure

The dominance of recall-failure errors from the early post-onset stage indicates an impairment in retrieving stored kanji representations from the visual orthographic lexicon. This pattern remained qualitatively stable despite quantitative improvement. Kana writing, which depends predominantly on phonological conversion routes, was preserved, whereas kanji writing, which relies on lexical-orthographic pathways, was selectively impaired, demonstrating a script-specific dissociation^7,8^.

Neuroimaging studies implicate the left posterior inferior temporal cortex and related networks in kanji writing and mental recall, and also suggest bilateral involvement in kanji lexical processing^7,15,16,17^. In this case, SPECT demonstrated hypoperfusion in the right occipito-thalamic region, which is consistent with possible dysfunction of homologous right-hemisphere visual-orthographic networks supporting access to kanji orthography.

The preserved ability to write kana and to copy visually presented forms argues against a global graphemic buffer or motor programming deficit; rather, the primary deficit appears to be access to stored kanji morphology. Our previous work has shown that kanji morphological knowledge is tightly linked to both writing and reading comprehension^12^, and the present case raises the possibility that such knowledge may, in some individuals, be partly supported by bilateral or right-biased networks in character-based writing systems.

The absence of alexia may be explained by the preserved function of the left VWFA and preserved reading pathways, despite impaired written output^18,19^. Thus, reading was maintained while writing was selectively impaired, consistent with reports of dissociation between reading and writing in kanji and morphogram processing^15,17^. A similar script-specific dissociation has been reported in crossed aphasia, in which kana reading was selectively impaired while kanji reading was preserved^16^. Although the direction of dissociation differs, this report supports the notion that logographic and phonographic processing need not break down through an identical mechanism even in crossed aphasia. Occasional orthographic and component errors may reflect secondary disturbances in reconstructing kanji morphology after retrieval failure. Similar orthographic-lexical impairment has also been described in left-hemisphere lesion cases^19,20,21,22^, suggesting that a comparable mechanism may arise in either hemisphere depending on lateralization.

### Comparison with previous reports

Kanji writing disorders after right-hemisphere damage have most often been reported as spatial or attentional agraphia, frequently associated with hemispatial neglect^9,10,11,13,14^, or as attention-related agraphia within broader visuospatial syndromes^23^. These cases typically show additions or omissions of radicals and strokes, misalignment within the character frame, or disorganized layout. By contrast, although the present patient exhibited left hemispatial neglect, kanji errors were clearly dominated by recall failures, whereas orthographic and component errors were only minor. In addition, in sentence dictation, the writing baseline was largely maintained horizontally, no definite rightward drift on the page was observed, and there were no systematic left-lateralized omissions of radicals/strokes. Taken together, the kanji agraphia in this case does not match the typical features of “classic” spatial agraphia and is less readily explained by spatial factors alone, instead suggesting a qualitatively distinct mechanism involving impaired access to the orthographic-lexical system.

Other right-hemisphere reports have attributed kanji agraphia to atypical language lateralization^9,10,24^, but their error profiles were largely visuospatial. In the current case, neglect and constructional disorder were present, yet the error pattern was recall-failure dominant, suggesting that the primary locus of impairment lies in the orthographic-lexical system rather than in spatial attention or construction. Kanji agraphia is more commonly described after left posterior temporal lesions^22^, often with combined orthographic errors and recall failures, interpreted as visual-motor conversion deficits affecting selection and execution of graphemic forms^18,19^. Reports of kanji-specific disorders with preserved kana^25,26^ resemble the present pattern, but have usually involved left-hemisphere lesions. Notably, a previous right-hemisphere case with kanji agraphia involved a family history of left-handedness^10^, whereas our patient was strongly right-handed with no such history and still developed crossed aphasia with kanji-specific recall failure. This finding is consistent with a possible contribution of right-hemisphere networks to kanji orthographic processing in some right-handed individuals.

In conclusion, this case shows that kanji-specific agraphia with an error profile dominated by recall failures can occur in crossed aphasia following a right posterior cerebral artery (PCA) infarction, and that its qualitative error profile may remain stable over at least one year despite partial gains in accuracy. The findings are consistent with a contributory role of right-hemisphere occipitothalamic networks in accessing the visual orthographic lexicon for kanji in some right-handed Japanese speakers. Clinically, they underscore the importance of detailed analysis of error types and longitudinal follow-up of kanji writing, even when overall aphasia is mild and kana writing appears intact, to detect and monitor persistent agraphia with an error profile dominated by recall failures linked to atypical hemispheric lateralization.

## Data Availability

The datasets generated and/or analyzed during the current study are not publicly available due to privacy and ethical restrictions but are available from the corresponding author upon reasonable request.
